# Incidence and predictors of diabetic ketoacidosis among children with diabetes in west and east Gojjam zone referral hospitals, northern Ethiopia, 2019

**DOI:** 10.1186/s13052-020-00930-4

**Published:** 2020-11-03

**Authors:** Birtukan Assefa, Haymanot Zeleke, Rajalakshmi Murugan, Kalkidan Wondwossen

**Affiliations:** 1 College of Health Sciences, Debre-Markos University, Debre-Markos, Ethiopia; 2 School of Nursing and Midwifery, College of Allied Health Sciences, Addis Ababa University, Addis Ababa, Ethiopia

**Keywords:** Diabetic ketoacidosis, Incidence, Diabetic mellitus, And children

## Abstract

**Background:**

Recurrent diabetic ketoacidosis in patients with known diabetes mellitus remains a relevant problem in pediatrics with an incidence of 1–10% per patient. Children may die because of cerebral edema and had a significant mortality (24%) and morbidity (35%).

**Objective:**

We assessed the incidence and predictors of diabetic ketoacidosis among diabetes children at East and West Gojjam zone referral hospitals, North West Ethiopia, 2019.

**Methods:**

An institution-based retrospective follow up study was conducted on children who were registered from January 1, 2014, to January 1, 2019. Epi data version 3.1 & Stata 14 were used for data entering and analysis respectively.

**Result:**

Out of 354 children included in the study, 207 (58.5%) developed diabetic ketoacidosis. The overall incidence rate of diabetic ketoacidosis was 2.27/100 children/month of observation. Age < 5 years (AHR: 3.52, 95% CI (2.25, 5.49), non-adherence (AHR: 1.54, 95% CI (1.11, 2.14), inappropriate insulin storage (AHR: 1.36, 95% CI (1.008, 1.85), presence of upper respiratory tract infections during diabetic ketoacidosis diagnose (AHR: 2.22, 95% CI (1.11, 4.45) and preceding gastroenteritis (AHR: 2.18, 95% CI (1.07, 4.44) were significant predictors.

**Conclusion:**

Age < 5 years old, non-adherence, inappropriate insulin placement at home, preceding gastroenteritis, and presence of upper respiratory tract infections at the time of diabetic ketoacidosis development were significant predictors. Hence, assessing and close monitoring as well as strengthened diabetic education should be given for the above predictors.

## Background

Diabetic Ketoacidosis (DKA) represent a state of acute metabolic stress, results when the body suffers due to an absolute or relative insulin deficiency for the metabolism of glucose [1]. Globally, recurrent-DKA in patients with known diabetes mellitus (DM) remains a relevant problem in pediatrics. According to the international society of pediatric and adolescent diabetes, the risk of DKA in known diabetes is 1–10% per patient per year in children [2]. The incidence of recurrent DKA is varied across the world due to the difference in the quality of health care services and socioeconomic circumstances. The incidence of DKA in known diabetes children in the US was 8 per 100 person-years [3], in Sweden 3.2–3.6/100 patient-years [4], in French 0.7% [5], in Indonesia 41.4% [6], in Germany and Austria 6% [7] and in Italy 38.5% [8]. Whereas in Africa the data on the incidence of DKA in known diabetes is scarce, but some studies report a high frequency of DKA in Sudan 92.1% [9], north-western Nigeria 62.2% [10], in South Africa 69.8% [11]. In the same way, in Ethiopia, to our knowledge the incidence of DKA in known diabetes is unstudied, rather a study in Addis Ababa showed that the prevalence of DKA at diagnosis of DM is 35.8% [12].

DKA is the most common cause of morbidity and mortality among children. It increases the risk of cerebral edema and cognitive deficits [13, 14]. A study showed that the risk of developing cerebral edema was 12.4 per 1000 episodes of DKA, which was higher than non-DKA DM patients (3.8 per 1000). It has a significant mortality (24%) and morbidity (35%) [15]. On the top of that, DKA allows a crisis in terms of health care costs and missed work, and school-days. One DKA-related hospital admission ranges from US$4125 to US$11196 costs [16]. The risk of DKA in known diabetes increased in patients with younger age (< 5 yrs.), infection, insulin omission, lower socioeconomic status, and lower parental education [17]. Despite an increase in diabetes in our country, there is limited study in Ethiopia on the incidence and predictors of diabetic ketoacidosis in children thus; the study aims to fill this gab.

## Methods and materials

### Study setting

The study was conducted in the two referral hospitals (Debre-Markos referral hospital and Felege-Hiwot referral hospital) of East and West Gojjam zones in Amara regional state North West Ethiopia. These hospitals serve more than 3.5 million and 5 million population in their catchment area respectively. Apart from other services, both referral hospitals offer diabetic treatment services.

### Study design

Five years of institution-based retrospective follow up study was conducted.

### Inclusion and exclusion criteria

Children age less than 15 years old and diagnosed with DM and having follow up care from January 1, 2014, to January 1, 2019, were included and the child who was developing DKA at the first diagnosis of DM and charts which was lost during the study period was excluded from the study.

### Data collection

Initially, we assessed the total DM caseload in the database on the registered follow-up chart/form from the discharge catalog of admitted patient’s pediatric ward, emergency and outpatient department from January 1, 2014, to January 1, 2019. Then the medical registration numbers of all diabetic pediatric patients were sorted. After this, a simple random technique was applied to select the required sample size of 376 children with diabetic. Finally, trained BSC nurses working at diabetic clinics collected the data from registered patient charts in the hospital by using a checklist that measures the socio-demographic, clinical, treatment characteristics and information on glycemic control of the children.

### Outcome measures

DKA was considered, in the context of hyperglycemia (Blood glucose measurement > 200 mg/dl or > 11 mmol/L) and any of the following present: a blood bicarbonate level < 15 mmol/L, and/or a pH < 7.30, and/or a DKA diagnosis mentioned in the medical records and/or Ketone body in the urine [2]. Appropriate insulin storage was considered in patients who store insulin in a refrigerator between 2 to 8 degree *Celsius* and keep away from heat and light. If a refrigerator is not available appropriate insulin storage was considered in patients who kept Vials at room temperature (20 to 25 degree *Celsius*) and protected from sunlight and heat for a maximum of 6 weeks, and 4 weeks (if the temperature goes up to 30 degree *Celsius* or within hot seasons) after initial use, in a clean plastic box (plastic container with cotton) [18].

### Ethical consideration

After the approval of the proposal, ethical clearance was obtained from the school of nursing and midwifery, college of health sciences, Addis Ababa University. Then permission letter was written to Debre Markos and Felege Hiwot referral hospitals to collect the data. We had taken permission from hospital medical directors and data was kept confidential. Informed consent was not required, due to data was taken from chart review only.

### Statistical analysis

The collected data were coded and entered into Epi data version 3.1 and cleaned and transferred to Stata version 14 for further analysis. The incidence rate of DKA was estimated per 100 DM children per month. The Kaplan Meier estimator was applied to estimate, the median time to develop DKA during the treatment period and log-rank tests, to compare survival curves. The predictors of DKA were analyzed by the Cox proportional hazard model with hazard ratio, 95% CI. The statistical test was considered significant at a *P* value of less than 0.05. Covariates and proportional hazard assumptions were checked using a log-log plot and goodness of fit by Schoenfeld residual test.

## Results

### Socio-demographic characteristics

Out of 376 children’s clinical profile reviewed, 354 were enrolled in the study. The rest of sample 22 (5.8) was incomplete data. From 354 children, more than half 159 (55.1%) were males and more than half 189 (53.4) of them were from a rural area. The mean age of the children at the time of DM diagnosis was 8.21 years with SD ±3.94 years (Table 1).

**Table 1:** Socio-demographic characteristics of DM diagnosed children at East &West Gojjam Zone referral hospitals, Northwest Ethiopia, 2019

Variable		Frequency N(354)	Percent %
Sex	Male	195	55.1
	Female	159	44.9
Residence	Urban	165	46.6
	Rural	189	53.4
Age	< 5 year	93	26.3
	5 -9 year	116	32.8
	> = 10 year	145	41.0

### Clinical characteristics

The majority of the 295 (83.3%) of children have normal weight for age. Around 258 (72.9%) have no family history of DM. The majority of children 317(89.5%) were diagnosed with type 1 DM, the rest were type 2 DM. About one-third of participants 119(33.6%) have got preceding infection; of which 346(97.7%) had upper respiratory tract infection (URTI) followed by 53 (15%) skin fungal infection including (tinea capitis 46(86.8%), Tinea corporis 5(9.4%) and cutaneous candidiasis 2(3.8%)) and 19(5.4%) pneumonia (Table 2).

**Table 2:** Clinical characteristics of DM diagnosed children at East &West Gojjam Zone referral hospitals, Northwest Ethiopia, 2019

Variable		Frequency (354)	Percent %
**Weight for age**	Normal	295	83.3
	Underweight	58	16.4
	Overweight	1	0.3
**Weight for height**	Normal	306	86.4
	Underweight	48	13.6
**Preceding infection**	Fungal skin infection	53	15
	Pneumonia	19	5.4
	Tuberculosis	3	0.8
	Tonsillitis	5	1.4
	Gastroenteritis	15	4.2
	Urinary tract infection	14	4
	Hepatitis	5	1.4
	Otitis media	7	2
	Chickenpox	2	0.6
	Meningitis	1	0.3
	Upper respiratory tract infection	346	97.7
	Other	18	5.1

**Fig. 1:**
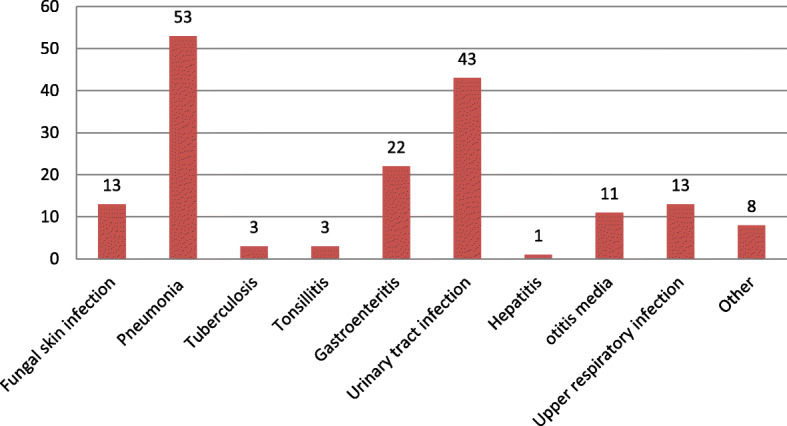
Present acute illness at the time of DKA diagnose children at East &West Gojjam zone referral hospitals, Northwest Ethiopia, 2019

**Fig. 2:**
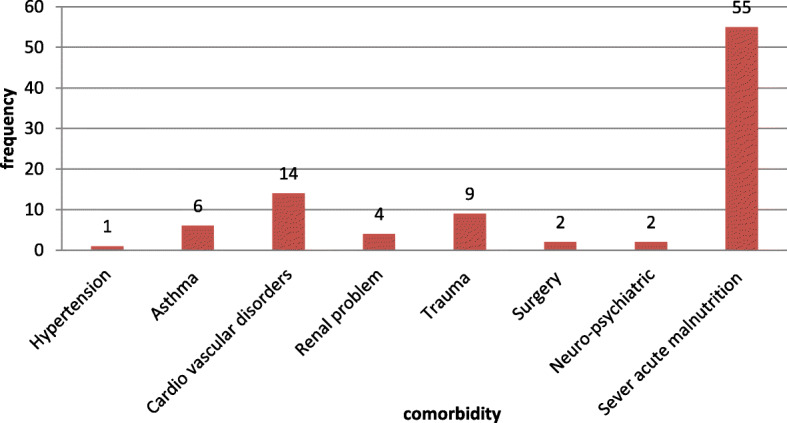
Comorbidities among DM diagnosed children at East &West Gojjam zone referral hospitals, Northwest Ethiopia, 2019

Nearly one-third of children 120 (33.9%) had an acute illness at the time of DKA development. Of which 53 (14.97%) had pneumonia followed by urinary tract infection (UTI) 28 (7.9%) and gastroenteritis 22 (6.2%) (Fig. 1). About 92 (26.0%) of children had comorbidity of which 55 (15.5%) had severe acute malnutrition (SAM) (Fig. 2) and 105 (29.7%) of children were hypoglycemic, 4 (1.1%) had acute kidney injury and 1 (0.3%) had chronic kidney injury after starting to follow up for DM.

### Treatment-related variables of DM diagnosed children

A diabetes care team is simply composed of a physician/general practitioners, and one compressive nurse and type of diabetes teaching/education given in our setting is one-on-one training is given in the hospital by nurses at their first diagnosis of diabetes on nutritional management, insulin injection techniques, exercise, and self-monitoring of blood glucose. One-fourth 171 (48.3%) of the children had a history of medication adherence and about 333 (94.5) children used insulin and 8 (2.3%) used hypoglycemic agents for treatment, but, about 13 (3.7%) not take any drug. About 247 (69.77%) children stored insulin appropriately at home and about three-fourth 265 (74.9) of the children had poor glycemic control.

### Incidence of diabetic ketoacidosis after DM diagnosis

**Fig. 3:**
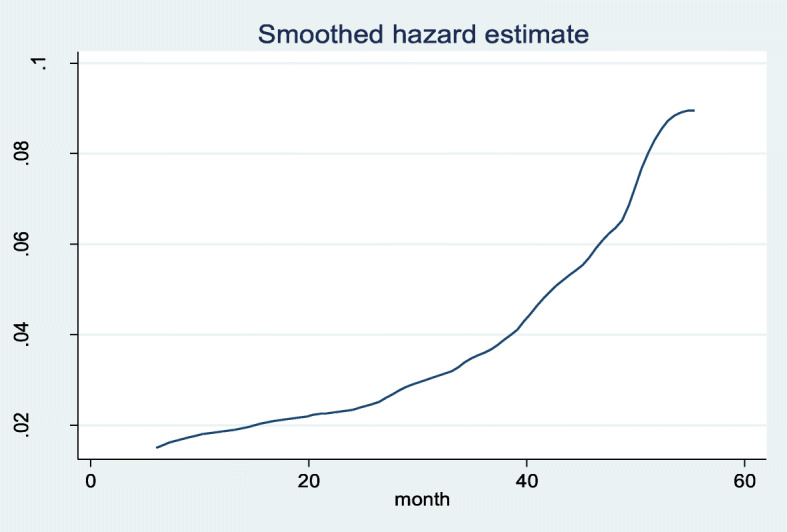
The hazard estimate of DKA after DM diagnosed children at East &West Gojjam zone referral hospitals, Northwest Ethiopia, 2019

Out of 354 children enrolled, 207 (58.5%) were developed DKA, with a mean follow up time of 25.72 months with 95% CI (24.1, 27.43). The children have followed a minimum of 1 month and a maximum of 5 years. The incidence rate of DKA was calculated using cases/month as a denominator for the entire cohort. The overall incidence rate of DKA in the cohort was 2.27 cases per 100 children per month. The median survival time of the entire cohort was found to be 35.6 months (IQR: 18.6, 49.2). When time is gone the hazard of developing DKA is going to high which is well described through hazard estimate (Fig. 3).

### Long rank test to compare survival curves

The test statistics showed that there was a significant difference in survival function for different categorical variables. These variables include age, family history, missed follow up, from preceding infection; tonsillitis, gastroenteritis, and meningitis, from acute recent illness at the time of DKA; upper respiratory tract infection, pneumonia, tonsillitis, gastroenteritis, and otitis media, children with severe malnutrition, types of drug used, medication adherence and insulin storage at home.

**Fig. 4:**
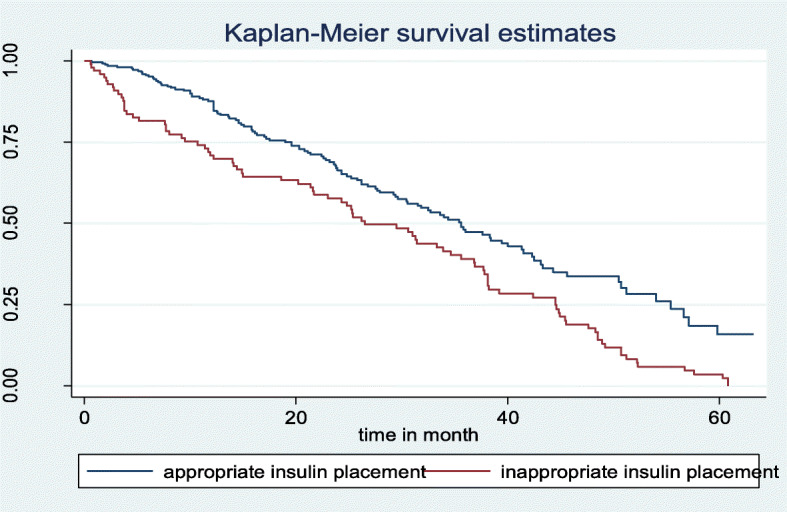
Kaplan-Meier survival estimate of DKA occurrence based on insulin placement at home at East &West Gojjam zone referral hospitals, North West Ethiopia, 2019

**Fig. 5:**
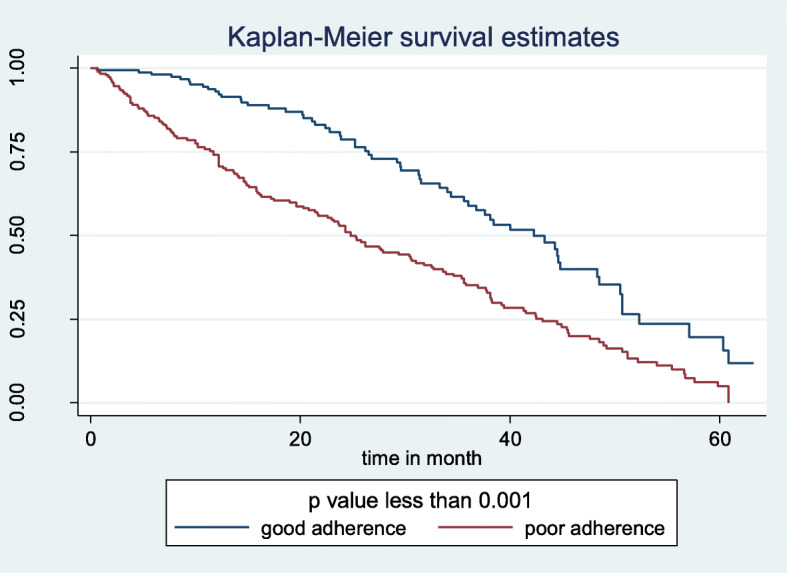
Kaplan-Meier survival estimate of DKA occurrence based on medication adherence at East &West Gojjam zone referral hospitals, North West Ethiopia, 2019

The median survival time for those having a history of inappropriate insulin storage at home was 33.3 months with CI (25.3, 38.1) and the median survival time for those who had a history of appropriate insulin storage at home was 35.8 months with 95% CI (30.5, 42.3). The survival time difference between the groups was found statically significant with a *P*-value of 0.0002 (Fig. 4). In addition, the median survival time for those who had a history of medication adherence was 44.3 months with 95% CI (36.9, 50.5) and the mean survival time for those who had a history of medication non-adherence was 27.5 months with 95% CI (23.6, 33.9). The survival time difference between the groups was found statically significant with *P* < 0.001 (Fig. 5).

### Predictor of diabetic ketoacidosis

The final multivariate cox proportional hazard adjusted model *revealed* that, the hazard of diabetic ketoacidosis decreased by 13.4% as age increased by 1 year (95% CI (2.34, 5.709). The hazard of Diabetic ketoacidosis was 3.52 times in children age < 5 years than those aged > 10 years (95% CI (2.25, 5.49). Also, the hazard of DKA in children who have preceding gastroenteritis was 2.18 times more than those who have no preceding gastroenteritis (95% CI (1.07, 4.45). Similarly, the hazard of DKA in children who have an upper respiratory tract infection at the time of DKA development was 2.22 times more than those who have no respiratory tract infection at the time of DKA development 95% CI (1.109, 4.45). The hazard of DKA was 1.36 times more in children who have a history of inappropriate insulin storage at home than those children who had a history of appropriate insulin storage at home 95% CI (1.008, 1.85). Lastly, the hazard of DKA in children who have a history of medication non-adherence was 1.54 times more than in children who have a history of medication adherence 95% CI (1.11, 2.14) (Table 3).

**Table 3:** Cox regression analysis for predictors of DKA

Variable			Survival status		CHR (95% CI)	***P***-value	AHR (95% CI)
			Event	Censor			
**Age**	< 5		59	34	4.58 (3.07, 6.82)	< 0.001*	3.52 (2.25, 5.49)
	5–10		87	61	1.76 (1.18, 2.62)	0.065	1.47 (0.97, 2.24)
	> 10		61	52	1		1
**Sex**	Male		111	84			1
	Female		96	63	0.83 (0.6, 0.097)	0.185	0.82 (0.61,1.09)
**Missed follow up**	Yes		88	48			
	No		119	99	0.75 (0.57, 0.99)	0.172	0.81 (0.61,1.09)
**Insulin storage**	Appropriate		105	142	1		1
	Inappropriate		102	5	1.98 (1.51, 2.62)	0.044 *	1.36 (1.008,1.85)
**Adherence**	Yes		61	110	1		1
	No		146	37	2.18 (0.95, 2.95)	0.01*	1.54 (1.11,2.14)
**Recent acute illness**	Pneumonia	Yes	50	3	0.62 (0.42, 0 .91)	0.615	1.09 (0.75,1.59)
		No	157	144	1		1
	upper respiratory tract infection	Yes	13	–	2.44 (1.39, 4.31)	0.024*	2.22 (1.109, 4.45)
		No	194	147	1		1
	Tonsillitis	Yes	3	–	3.06 (0.97, 9.64)	0.800	1.29 (0.174, 9.60)
		No	204	147	1		
	Gastroenteritis	Yes	20	2	1.69 (1.06, 2.68)	0.907	1.035 (0.57,1.87)
		No	187	145	1		
	urinary tract infection	Yes	43	–	1.61 (1.15, 2.25)	0.923	1.73 (0.67,1.41)
		No	164	147	1		1
	otitis media	Yes	11	–	1.98 (1.08, 3.65)	0.194	1.54 (0.801,2.92)
		No	196	147	1		1
	skin fungal infection	Yes	13	–	1.67 (0.95, 2.94)	0.096	1.73 (0.90,3.30)
		No	194	147	1		
Preceding infection	tuberculosis	Yes	3	–	2.91 (0.92, 0.16)	0.071	3.7 (0. 89–15.85)
		No	204	147	1		1
	Tonsillitis	Yes	5	–	2.67 (1.09, 6.52)	0.498	1.82 (0.32,10.39)
		No	202	147	1		1
	gastroenteritis	Yes	12	3	2.27 (1.26, 4.09)	0.032*	2.18 (1.07, 4.46)
		No	195	144	1		1
	urinary tract infection	No	11	3	1.68 (0.91, 3.09)	0.189	1.58 (0.79, 3.15)
		Yes	196	144	1		1
	Meningitis	Yes	1	–	8.28	0.316	0.18 (0.006, 5.13)
		No	206	147	1		1
	other infections	Yes	12	6	1.41 (0.78, 2.54)	0.446	1.27 (0.68, 2.08)
		No	195	141	1		1
Having sever acute malnutrition		Yes	42	13	1.41 (1.004,1.98)	0.901	1.02 (0.69, 1.50)
		No	165	134	1		1

Keynote * variables which were significant at *p*-value < 0.05 and - mean 0 observation

## Discussion

At the end of 5 years follow up, about 207 (58.4%) developed DKA with the overall incidence of 2.27 cases per 100 children per month (27.24 cases per 100 child-years) observation. The incidence rate of DKA in this study is higher than studies done in the US which were 8 per 100 person-years [3], in Sweden 3.2–3.6/100 patient-years [4], the international society of pediatric and adolescent diabetes 2014 report 1–10% per patient per year [2] and Austria 8.4 to 18.4 per 100,000 per year [19]. Regarding cumulative incidence, this finding is consistent with studies done in north-western Nigeria which was 62.2% [10] and 55.5% in Iran [20]. However, this finding is much higher than studies done in the US which was 25.5% [21], 38.5% in Italy [8], 41.4% in Indonesia [6], 28% in Poland [22], 40% in southern Iraq [23]. This discrepancy might be due to difference in methodology, lifestyle, culture, economic status, access to health care facilities, and level of education of the general public.

Lack of appropriate patient (and family) education concerning the home self-management [24] may have contributed to increased incidence of DKA in Ethiopia. Thus, insufficient education and resources about self-monitoring and DKA prevention can have a great impact on DKA existence in many patients and contribute to most of the increased morbidity and premature mortality [25, 26]. Furthermore, poor access to health care facilities in our country [27] accompanies many patients to seek alternative treatments such as consulting traditional healers, using herbal remedies [28] prayers and rituals that encountered a delay in care which further complicating the disease process [29].

Children with age < 5 years were more likely to develop DKA compared to age > 10 years old. This is consistent with other previous studies conducted in the US [21], Italy [8], Southern Iraq [23]. This might be children with age < 5 -year might be more dependent on their caregiver and more venerable to medication non-adherence. Also, this age group can encounter trouble for lifestyle modifications which are the backbone for preventing the occurrence of DKA such as adhere to a diet, exercise, and self-monitoring of blood glucose level. Also, in our study, children who have medication non-adherence were more likely to develop DKA as compared to children who adhere to medication. This finding is supported by other previous studies conducted in sub-Saharan Africa [30], north India [31], Saudi Arabia [32], Southern Iraq [23]. This might be since DM is a chronic illness after taking medication symptoms may disappear for some time so; the children may not take their medication on time.

In this study child having a history of inappropriate insulin storage at home were more likely to develop DKA. This might be most of the participants were from rural areas and may not have appropriate storage materials like refrigerators. Besides, because of our poor diabetic education services [24] they may have inadequate knowledge about insulin storage during temperature variation and duration of storage. Lastly, in this study, children who have preceding gastroenteritis and upper respiratory tract infection at the time of DKA development were more likely to develop DKA as compared to those who have not. This is supported by the study, in Nigeria [10], sub-Saharan Africa [30], Malaysia [33], north India [31], and Saudi Arabia [32]. This might be infection can cause excessive levels of counteracting hormones, mainly cortisol and adrenaline, which triggering an episode of DKA. When there is gastroenteritis, there will be vomiting and diarrhea-causing dehydration and electrolyte imbalance. This causes an increase in the stress hormone.

### Limitations

Since the data were collected from medical records, patients’ charts lost and incomplete data were found. These may affect the outcome of the study. Also, this study did not include the recurrence of diabetic ketoacidosis (trend) and the lack of some variables like parental factors that can’t be addressed through chart review.

## Conclusion

In conclusion, the incidence of DKA in known diabetes children found to be high. Children who have age < 5-year, medication none adherence, inappropriate insulin placement at home, presence of upper respiratory tract infections at the time of DKA development, and presence of preceding gastroenteritis were predictors of DKA development at East and West Gojjam zone referral hospitals, Northwest Ethiopia. Therefore, Diabetic care clinics need to be strengthened. Diabetic clinic care should focus on assessing, close monitoring and enhancing diabetic education for patients and caregiver/families/ to over control the predicators of DKA. Finally, we recommend those variables that cannot be assessed through card review and recurrence DKA will be investigated with another study design.

## Data Availability

All materials and data are available from the corresponding author without any restriction.

## Abbreviations

AHR: Adjusted hazard ratio
CHR: Crude hazard ratio
DM: Diabetic mellitus
DKA: Diabetic ketoacidosis
COPD: Chronic obstructive lung disease
HTN: Hypertension
SD: Standard deviation
IQR: Interquartile range
CI: Confidence interval
US: United States
URTI: Upper respiratory tract infection
UTI: Urinary tract infection
SAM: Severe acute malnutrition
